# Qualitative and Quantitative Saponin Contents in Five Sea Cucumbers from the Indian Ocean

**DOI:** 10.3390/md8010173

**Published:** 2010-01-21

**Authors:** Séverine Van Dyck, Pascal Gerbaux, Patrick Flammang

**Affiliations:** 1 Marine Biology Laboratory, University of Mons, 20 Place du Parc, B-7000 Mons, Belgium; E-Mail: Severinevandyck@hotmail.com (S.V.D.); 2 Organic Chemistry Laboratory, Mass Spectrometry Center, University of Mons, 20 Place du Parc, B-7000 Mons, Belgium

**Keywords:** Holothuriidae, triterpene glycosides, tandem mass spectrometry, hemolysis, orcinol reaction

## Abstract

To avoid predation, holothuroids produce feeding-deterrent molecules in their body wall and viscera, the so-called saponins. Five tropical sea cucumber species of the family Holothuriidae were investigated in order to study their saponin content in two different organs, the body wall and the Cuvierian tubules. Mass spectrometry techniques (MALDI- and ESI-MS) were used to detect and analyze saponins. The smallest number of saponins was observed in *Holothuria atra*, which contained a total of four congeners, followed by *Holothuria leucospilota*, *Pearsonothuria graeffei* and *Actinopyga echinites* with six, eight and ten congeners, respectively. *Bohadschia subrubra* revealed the highest saponin diversity (19 congeners). Saponin mixtures also varied between the two body compartments within a given animal. A semi-quantitative approach completed these results and showed that a high diversity of saponins is not particularly correlated to a high saponin concentration. Although the complexity of the saponin mixtures described makes the elucidation of their respective biological roles difficult, the comparisons between species and between body compartments give some clues about how these molecules may act as predator repellents.

## 1. Introduction

Holothuroids, also known as sea cucumbers, are marine animals that are characterized by a slow motion and the absence of prominent structural defenses. As a direct consequence, they are vulnerable to predation. As a mean of defense, most sea cucumbers contain, in their body wall and viscera, secondary metabolites named saponins [1–4]. Their structures are based on a lanosterol-type triterpene with a distinctive D-ring with fused γ-lactone skeleton named holostane and a carbohydrate chain containing up to six sugar residues such as glucose (Glc), 3-*O*-methylglucose (MeGlc), quinovose (Qui), and xylose (Xyl) [5,6]. In addition, in some species, sulfate groups may be present at certain positions of the sugar units [7]. Because these compounds possess a large spectrum of pharmacological activities [8], numerous studies are being currently conducted to identify new congeners and new natural sources of saponins.

On the other hand, the biological roles of saponins in holothuroids are still very speculative [9,10]. They are abundant in the body wall which, in addition to its role as a physical barrier protecting the animal, is also the largest organ [3,11,12]. They also appear to be particularly concentrated in the Cuvierian tubules, a specialized defense system developed by some sea cucumber species, all belonging to the family Holothuriidae [3,4,11]. This organ, located in the posterior part of the animal, consists of multiple tubules that can be expelled by the individual after stimulation [13,14]. Expelled tubules lengthen into sticky white threads that may entangle potential predators. To date, however, only a few authors have investigated the qualitative and quantitative differences between body wall and Cuvierian tubule saponins (see e.g., Kobayashi *et al*. [11]).

Recent studies have demonstrated that mass spectrometry (MS) procedures represent very valuable techniques for the detection and identification of saponins [15–17]. Indeed, using MS methods such as MALDI-MS (Matrix-Assisted Laser Desorption/Ionization-Mass Spectrometry) and LC-MS (Liquid Chromatography-Mass Spectrometry) techniques, we were able to highlight remarkable differences between the saponin mixtures from the body wall and the Cuvierian tubules of *Holothuria forskali* [18]. In this species, which was first studied by Rodrigez *et al*. [19], only five saponins were described, with no indication on their organ of origin. Using MS, we detected 12 different saponins in the body wall and 26 in the Cuvierian tubules and highlighted the occurrence of many isomer congeners [18]. In continuation of our study on triterpene glycosides from sea cucumbers of the family Holothuriidae, we carried out a large-scale comparative investigation on saponins from five tropical species from the Indian Ocean. MS methods were used to detect and analyze saponins and a semi-quantitative study was also performed to compare total saponin contents. A peculiar attention was paid to the differences that could occur between the body wall and the Cuvierian tubules in a given species.

## 2. Results

### 2.1. Qualitative study

Saponins of the five species have been extracted and analyzed using MALDI-MS/MS and LC-MS/MS. MALDI-MS technique was used for direct detection and analysis of saponin mixtures, while the LC-MS technique was performed to achieve chromatographic separation of potential isomers (see Supplementary Figure 1 for a typical example). Indeed, in a previous work, we highlighted the presence of isomers in such saponin mixtures [18]. The entire extraction and purification procedures and the mass spectrometric analyses were the same as those used in Van Dyck *et al*. [18].

**Figure 1:**
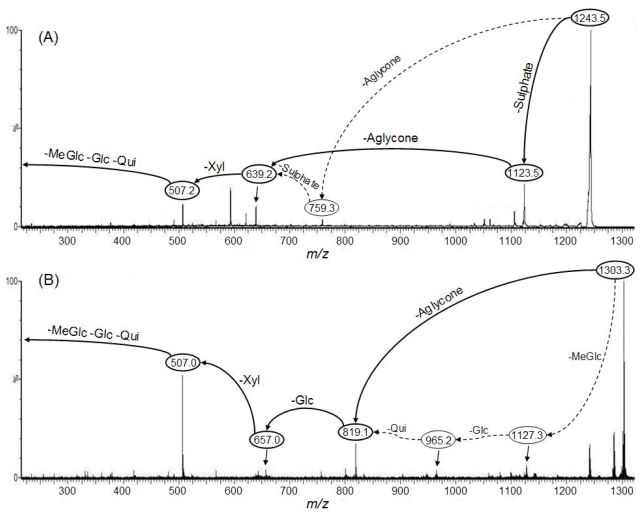
Comparison between the collision-induced fragmentation patterns of holothurin A (A) and holothurinoside A (B). Full and dotted arrows are two possible fragmentation patterns (see Figure 2 for molecular structures of these saponins and their respective fragments).

**Figure 2:**
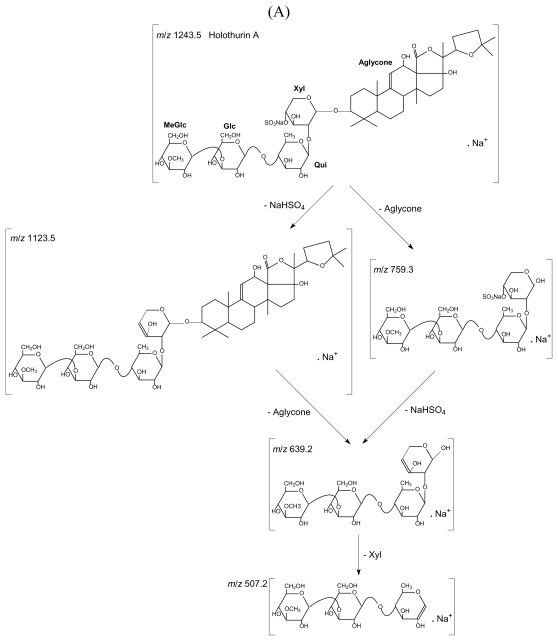

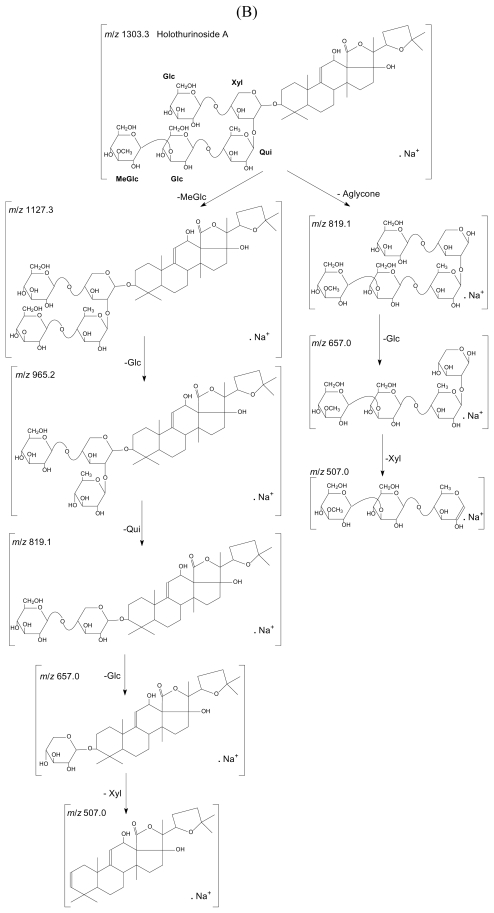
(A) Collision induced dissociation of *m/z* 1243.5 cations corresponding to holothurin A. (B) Collision induced dissociation of *m/z* 1303.3 cations corresponding to holothurinoside A. Glc: glucose, MeGlc, 3-*O*-methylglucose, Qui: quinovose, Xyl: xylose.

As a characteristic example, Figure 1 presents the tandem MS spectra of holothurin A (**1**) and of holothurinoside A (**10**) ions. It is noteworthy that the observed ions arise from cationization (Na^+^ attachment) of the neutral molecules upon ESI or MALDI. Based on MS/MS spectra and as described in detail in Van Dyck *et al*. [18], the molecular structures of the saponins can be obtained by the identification of the mass transitions between the successive collision-induced fragmentation peaks. For instance, in Figure 1A, two competitive sequences of decompositions are represented by arrows showing the consecutive losses of sodium monohydrogenosulfate (NaHSO_4_), of the aglycone, of xylose, and of quinovose, glucose and 3-*O*-methylglucose from the mass-selected holothurin A (**1**) ions (*m/z* 1243.5). This sequence of fragmentation is exemplified in Figure 2A. Similarly, Figure 1B is characterized by the consecutive losses of the aglycone, glucose and xylose, and, competitively, of quinovose, glucose and 3-*O*-methylglucose (see also Figure 2B). The detection of this sequence of fragmentations allows the identification of the *m/z* 1303.3 ions as cationized holothurinoside A (**10**).

The main difference between both MS/MS spectra lies in the presence of a sulfate group in holothurin A (**1**). The presence of a sulfate group was confirmed (Figure 1A) by the occurrence of a fragment ion detected at *m/z* 1123.5 that corresponds to ions obtained by a 120 amu (atomic mass unit) loss (NaHSO_4_) from the parent ions. This loss of 120 amu from the parent ion will definitively be the signature of the presence of a sulfate group on the saponin. On the other hand, both MS/MS spectra share the common *m/z* 507 key signal as a signature of the MeGlc-Glc-Qui oligosaccharide chain, actually [MeGlc-Glc-Qui + Na^+^] [18].

**Figure 3:**
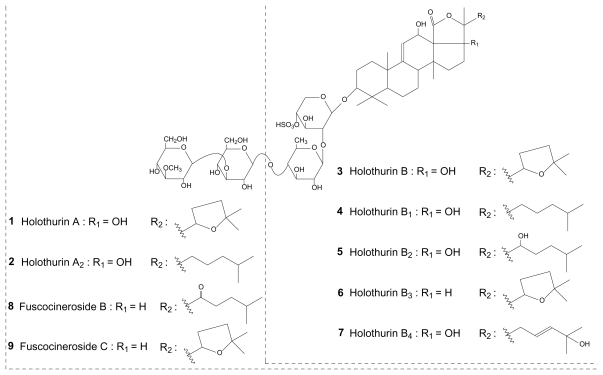
Molecular structures of holothurins and fuscocinerosides.

**Figure 4:**
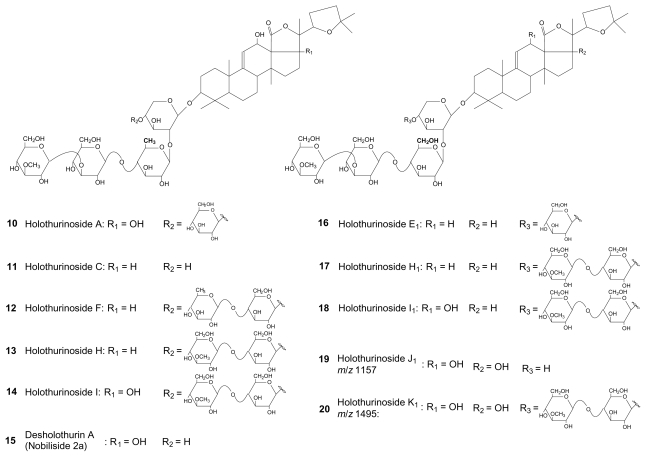
Molecular structures of holothurinosides, desholothuin A and proposition of structure for the saponins detected at *m/z* 1157 and 1495 (respectively named holothurin osides J_1_ and K_1_).

**Figure 5:**
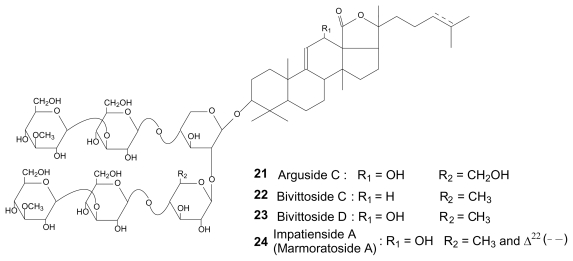
Molecular structures of arguside C, bivittosides C and D and impatienside A (marmoratoside A).

Similar mass spectrometric analyses were performed for all the body wall and Cuvierian tubules saponin extracts from the five investigated species. For each analysis, fragmentation patterns were built and saponin structures were elucidated or proposed. Indeed, in most of the cases, a literature survey allowed us to identify the saponins. When identification was not possible, molecular structures were tentatively proposed. Nevertheless, some isomeric saponins could not be identified and, in such cases, only molecular masses are reported as a support for further investigations. Table 1 summarizes the results of these analyses and the identified and proposed molecular structures are presented in Figures 3 to 5.

Table 1 highlights that each species and, within each species, each body compartment presents its own saponin mixture. Several saponins are widely represented among species, as holothurin A (**1**) and desholothurin A (**15**), compared to others which are scarcely represented (e.g., the saponin detected at *m/z* 1495 (compound **19**) which was only detected in *B. subrubra*). On some occasions, the methodology used was not adequate to unambiguously identify the molecular structures and several possibilities were offered by the literature. Indeed, differences between isomeric saponins are not always detectable by the used MS methodology that only relies on low-kinetic energy collision-induced dissociations (low energy CID). For example, it was the case for the saponin detected at *m/z* 1227 which could correspond to fuscocineroside B (**8**) or C (**9**) (Figure 3), the two molecules differing only at the level of the lateral chain of the aglycone. The same problem occurred for holothurins B (**3**) and B_4_ (**7**) (Figure 3). The complete structure elucidation of all the isomeric saponins detected by LC-MS was not possible for the same reasons.

The species *A. echinites* presented 10 sulfated saponins in the body wall, holothurins A, A_2_, B/B_4_, B_1_, B_2_, B_3_, fuscocineroside B/C and three isomers (Table 1), but only two in the Cuvierian tubules (holothurin A and fuscocineroside B/C). *B. subrubra* contained 12 non-sulfated saponins in each body compartment. Six of them are common to both organs (holothurinosides H, H_1_, I, I_1_, impatienside A (marmoratoside A) and an unidentified saponin detected at *m/z* 1495) while the others are organ specific: holothurinoside F, 4 isomers and an unidentified saponin detected at *m/z* 1157 in the body wall, and arguside C, bivittosides C, D and three isomers in the Cuvierian tubules. Structures have been tentatively proposed for the two unidentified saponins which have been named holothurinosides J_1_ and K_1_, respectively (**19** and **20** in Figure 4). *H. atra* enclosed 4 saponins, hothurins B/B_4_, B_1_, B_2_ and B_3_, all sulfated (Table 1). *H. leucospilota* and *P. graeffei* had both a large diversity of saponins including sulfated and non-sulfated ones. The two body compartments of *H. leucospilota* contained bivittoside D and holothurins B and B_3_, the Cuvierian tubules enclosing in addition desholothurin A (nobiliside 2a), holothurin A and holothurinoside E_1_. Finally, *P. graeffei* presented desholothurin A (nobiliside 2a), holothurins A, A_2_, B and fuscocineroside B/C in the body wall and the Cuvierian tubules. The former also contained holothurinoside C and one isomer, and the latter one isomer (Table 1).

### 2.2. Semi-quantitative study

A semi-quantitative approach has been realized in order to obtain data on the total saponin content of the different species. The aim of this approach was to quantify, for each body compartment and for each species, the concentration of its specific saponin mixture as a whole. Two complementary methods were used based on the structures and properties of saponins, respectively. On the one hand, the orcinol reaction evaluated the glycoside content of the different extracts; on the other hand, the measurement of the hemolytic activity of these extracts reflected the effectiveness of the saponin mixture to lyse the erythrocytes of cow blood. The spectrophotometric measurements obtained with these tests were converted respectively to milligrams of glycoside by gram of tissue and to milligram equivalents of plant saponins by gram of tissue, using the standard curves and the wet weights of the body compartments (Table 2).

Although the two methods do not measure the same characteristics of the sample, there was a significant correlation between their respective results (Pearson correlation test: r = 0.902, p < 0.001, n = 31). In many cases, the inter-individual variability was high (Table 2). However, proportionally, for a given species, the concentration of saponins was always higher in Cuvierian tubules than in the body wall. The concentration ratios between the two organs varied from 9 to 11-fold in *A. echinites*, 2 to 7-fold in *P. graeffei*, 1.5 to 2.5-fold in *B. subrubra* and 1 to 4-fold in *H. leucospilota* (Table 2). The Cuvierian tubules of *A. echinites* present the highest saponin content followed by those of *P. graeffei*, *B. subrubra* and *H. leucospilota*, irrespective of the method used. Results are more confused for body wall saponins. Differences between the five species being smaller in this body compartment, the two techniques gave slightly different rankings (Table 2).

## 3. Discussion

Five tropical species of sea cucumbers of the family Holothuriidae were inv estigated in order to study their saponin content in two different organs, the body wall and the Cuvierian tubules. After extraction and purification, saponins were detected using MS techniques (MALDI- and ESI-MS). These techniques corroborate literature data, confirming the existence and the structures of many saponins, but they also allowed the detection of several new saponin congeners in all five species. The smallest number of saponins was observed in *H. atra* which enclosed a total of four sulfated congeners. Among these, two were already described in the literature for this species [4,11,30]. Contrary to some of these authors, however, we did not detect holothurins A and A_2_ in *H. atra. B. subrubra*, on the other hand, revealed the highest saponin diversity (12 in each body compartment, all non-sulfated). To the best of our knowledge, nothing is reported in the literature on the saponins from this species. The body compartments of *A. echinites* contained ten sulfated saponins. In the literature, only four saponins were described for this species: holothurins A and B [4] and holothurins A_2_ and B_1_ (echinosides A and B in the text; [11,26,30]). Both *H. leucospilota* and *P. graeffei* contained mixtures of sulfated and non-sulfated saponins, representing a total of six congeners in the former and eight in the latter. Again, our results complete the literature since only holothurins A and B had already been detected in *H. leucospilota* [4,11,25,27,30,31] and holothurin A, A_2_ and B in *P. graeffei* [4,11,30]. These saponins are all sulfated. More recently, two new triterpene glycosides were discovered in *H. leucospilota* called leucospilotaside A and C [32,33], that we did not found in our samples.

In view of the obtained pieces of information, it appears relevant to classify saponins into two groups based on the presence or the absence of the sulfate moiety (sulfated *vs*. non-sulfated saponins). Amongst the investigated species, *A. echinites* and *H. atra* contain exclusively sulfated saponins while only non-sulfated congeners were detected in *B. subrubra*. This division is consistent with data reported in the literature showing that triterpene glycosides from the genus *Bohadschia* do not contain a sulfate group while those of the genera *Actinopyga*, *Holothuria* (except *H. forskali*; [18,19]) and *Pearsonothuria* possess such a group [4,6,11,30,34,]. However, according to our MS-based results, *H. leucospilota* and *P. graeffei* contain both types of saponins, even though sulfated saponins are the major congeners. This makes the situation more complex than it was previously thought. It might be interesting to investigate whether this distribution of sulfated and non-sulfated saponins follows the phylogeny of the family Holothuriidae.

A semi-quantitative study was also performed to highlight differences in saponin concentrations between body compartments and between species. However, the high variability in saponin content between different individuals from a same species makes inter-specific comparisons difficult. Moreover, for the body wall, the two methods used (hemolytic activity and orcinol reaction) did not give convergent results. For Cuvierian tubules, on the other hand, both methods indicate that triterpene glycosides would be more concentrated in *A. echinites*, followed by *P. graeffei* and *B. subrubra*, and finally by *H. leucospilota*. In addition, for all species, saponins were always more concentrated in the Cuvierian tubules than in the body wall. Only Matsuno and Ishida [3] compared the saponin content of the Cuvierian tubules to that of the body wall in the species *H. leucospilota*. The concentration ratios they reported (2 to 3-fold) are in the range we measured (1 to 4-fold). In our study, the highest ratio (11-fold) was observed in *A. echinites*. Species of the genus *Actinopyga* have long been known to possess saponin-rich Cuvierian tubules [35,36].

In addition to these quantitative differences, qualitative differences were also detected between the saponin mixtures of the two body compartments. Some species possess more saponin congeners in the Cuvieran tubules than in the body wall (*H. leucospilota*), some less (*A. echinites*), and some have roughly the same number of saponins in the two organs (*B. subrubra* and *P. graeffei*). However, the Cuvierian tubules of all species (except *A. echinites*) enclose at least one specific saponin. Similar differences in the body distribution of saponins have already been reported [11,18].

High saponin concentrations and specific congener mixtures are usually associated with the defensive function of Cuvierian tubules [10]. Yet, within the Holothuriidae, these organs differ greatly in terms of their morphology and their mode of functioning [14,37,38]. The most common tubule type consists of small white and smooth caeca, in the genera *Bohadschia, Holothuria* and *Pearsonothuria*. In the former two, these tubules are ejected through the anus when the animal is attacked, elongate in sea water, and become sticky upon contact with the predator which is then immobilized. In *Pearsonothuria graeffei*, the tubules are never ejected although they can be elongated manually and then become sticky. Finally, in the genus *Actinopyga*, the tubules are small and highly ramified and their surface is lobulated. These tubules are never ejected nor become sticky, and break very easily when stretched manually. The high saponin concentrations we measured in the Cuvierian tubules of *Pearsonothuria graeffei* and *Actinopyga echinites* suggests a shift from an adhesive-based defense mechanism to a toxicity-based mechanism in these species. In parallel, their tubules enclose mostly sulfated saponins that are more hydrophilic and could therefore diffuse in sea water at a higher rate (see also Kalinin *et al.* [10]). However, the type of Cuvierian tubules observed in *Actinopyga* would function as a defense mechanism provided only that the holothuroid is able to expose them to a potential predator. A behavior in which tubules are exposed through the anus without ejecting them and then are retracted into the body cavity has been described in species from the genera *Actinopyga*, *Bohadschia* and *Holothuria* [13,36,39].

Finally, there are several species in the genus *Holothuria* which completely lack Cuvierian tubules, making their importance of this organ as a defense system questionable. In this context, the comparison between *H. leucospilota* and *H. atra* is interesting. Indeed, these two species are sympatric, live in the same habitat (beneath and underneath the rocks on the reef crest) and present the same shape, size and color [40]. However, only *H. leucospilota* possesses Cuvierian tubules. In terms of concentration, there is no difference in the saponin content of the two species; *H. atra* does not appear to compensate its lack of Cuvierian tubules by a higher saponin concentration in its body wall. In terms of saponin diversity, on the other hand, the two species share only two congeners: holothurins B/B_4_ and B_3_. Contrary to *H. leucospilota* which enclose a mixture of sulfated and non-sulfated saponins, *H. atra* contains only sulfated triterpene glycosides. Sulfated saponins are considered to be more diffusible in sea water and more potent as toxins [10,41]. The highest number of non-sulfated saponins occurs in the Cuvierian tubules of *H. leucospilota*. According to Hamel and Mercier [13], Cuvierian tubules in this species are used as a dissuasive defense mechanism rather than as an aggressive weapon, their toxicity acting as a repellent when the tubules are tasted without representing a threat to the health of fish predators. Less soluble non-sulfated saponins would remain longer in the tubule tissue and thus increase their efficacy as a repellent.

This whole study of the saponin content of these five species allowed us to complete the data from the literature and to highlight the large number and the high diversity of saponins in the Holothuriidae. The semi-quantitative approach completed these results and showed that a high diversity of saponins is not especially correlated to a high concentration of saponin. Although the complexity of the saponin mixtures described makes the elucidation of their respective biological roles difficult, the comparisons between species and between body compartments gave some clues about how these molecules may act as predator repellents. Further works must be done on the specific tissular localization of saponins as well as on their effectiveness against predator at natural concentrations in order to confirm their biological role.

## 4. Experimental Section

### 4.1. Sampling

Specimens of *Actinopyga echinites* (Jaeger, 1833) and *Pearsonothuria graeffei* (Semper, 1868) were collected at depths ranging from 10 to 30 m by scuba diving at the south point of the Great Reef of Toliara (Madagascar). Individuals of *Bohadschia subrubra* (Quoy and Gaimard, 1833), *Holothuria atra* (Jaeger, 1833) and *H. leucospilota* (Brandt, 1835) were hand-collected at low tide on the crest of the Great Reef of Toliara. All the animals were transported to the “Institut Halieutique et des Sciences de la Mer” (IH.SM, Toliara), where they were kept in marine aquaria equipped with circulating sea water system (28°C, 31‰ salinity). Specimens were then dissected and body wall and Cuvierian tubules were stored separately in 70% ethanol at −20°C until used.

### 4.2. Extraction and purification of saponins

The extraction and purification procedures were adapted from Campagnuolo *et al.* [42] and Garneau *et al.* [43], and were described in detail in our previous paper [18]. Body wall and Cuvierian tubules underwent the same extraction method, which was usually repeated on several individuals. Briefly, the homogenized tissue was extracted twice with ethanol-water (70:30) followed by filtration. The extract was then evaporated at low pressure in a double boiler at 30°C using a rotary evaporator (Laborota 4001 efficient, Heidolph). The dry extract was diluted in 90% methanol and partitioned against n-hexane (v/v). The water content of the hydromethanolic phase was adjusted to 20% (v/v) then to 40% (v/v), these solutions being partitioned against CH_2_Cl_2_ and CHCl_3_, respectively. Finally, the hydromethanolic solution was evaporated and dissolved in water in order to undergo chromatographic purification. The crude aqueous extract was placed on a column of Amberlite XAD-4 (Sigma-Aldrich, St. Louis, MO). Washing the column with water removed the inorganic salts and subsequent elution with methanol allowed to recover saponins. The methanolic phase was then evaporated and the dry extract was diluted in water in order to undergo a last partitioning against *iso*-butanol (v/v). The butanolic fraction contained the purified saponins.

### 4.3. Mass spectrometry

All MS experiments were performed on a Waters QToF Premier mass spectrometer in the positive ion mode, either using the MALDI or the ESI source. The MALDI source was constituted by a nitrogen laser, operating at 337 nm with a maximum output of 500 mW delivered to the sample in 4 ns pulses at 20 Hz repeating rate. All samples were prepared using a 10 mg/mL solution of α-cyano-4-hydroxy-cinnamic acid in acetone as the matrix. The matrix solution (1 μL) was spotted onto a stainless steel target and air dried. Then, 1 μL of each butanolic fraction was applied onto the spots of matrix crystals, and air dried. Finally, 1 μL droplets of a solution of NaI (2 mg/mL in acetonitrile) was added to the spots on the target plate. Typical ESI conditions were: capillary voltage, 3.1 kV; cone voltage, 50 V; source temperature, 120°C; desolvation temperature, 300°C. Dry nitrogen was used as the ESI gas.

For the recording of the single-stage MALDI- or ESI-MS spectra, the quadrupole (rf-only mode) was set to pass ions between *m/z* 50 and 1500, and all ions were transmitted into the pusher region of the time-of-flight analyzer where they were mass-analyzed with a 1 s integration time. For the MALDI- or ESI-MS/MS CID experiments, the ions of interest were mass-selected by the quadrupole mass filter. The selected ions were then submitted to collision against argon in the T-wave collision cell (pressure estimated at 0.9 – 1 mBar) and the laboratory frame kinetic energy was selected to afford intense enough product ion signals. All the ions exiting the collision cell, either the product ions or the non dissociated precursor ions, were finally mass measured with the oa-ToF analyzer. Time-of-flight mass analyses were performed in the reflectron mode at a resolution of about 10,000.

For the on-line LC-MS/MS analyses, a Waters Alliance 2695 liquid chromatography apparatus was used. The HPLC device was coupled to the QToF Premier mass spectrometer (Waters) and consisted of a vacuum degaser, a quaternary pump and an autosampler. Sample volumes of 20 μL were injected. Chromatographic separation was performed on a non polar column (Symmetry C18, 4.6 × 150 mm, 5 μm, Waters) at 27°C. The mobile phase (1 mL/min) was a nonlinear gradient program from methanol (eluent A) and water (eluent B). The gradient program was: 10% of eluent A at start, 0–6 min 10 to 60% A, 6–13 min 60 to 95% A and 13–15 min back to 10% of eluent A. The mobile phase flow (1 ml/min) was splitted prior injection in the ESI source (200 μL/min).

### 4.4. Semi-quantitative study

Two complementary techniques, the measurement of the hemolytic activity and the estimation of the carbohydrate content with the orcinol reaction, were used to estimate the variability of the saponin content between species and between body compartments in a same species.

#### Hemolytic activity

The method was adapted from Kalinin *et al.* [44] and from Mackie *et al.* [45]. Citrated blood from cows was used in this experiment. Erythrocytes were pelleted by centrifugation at 1000 *g* for 15 min, and were washed and centrifuged three times in cold PBS buffer (140 mM NaCl, 2.7 mM KCl, 1.5 mM KH_2_PO_4_, 8.1 mM Na_2_HPO_4_·2H_2_O, pH 7.4) until the supernatant was clear and colorless. Two mL of packed cells were then diluted to 100 mL with the same buffer. Twenty microliters of each saponin extract was added to 1980 μL of the erythrocyte suspension in a microtube. The suspensions were mixed by inversion, incubated for 1 h at room temperature, and finally centrifuged. The extinction of the clear red supernatant was then measured at 540 nm using a Labsystems multiscan MS spectrophotometer. A 10 mg/mL solution of plant saponins (saponins from *Quillaja* bark; S 4521, Sigma-Aldrich, St. Louis, MO) in PBS was serially diluted to make a standard curve.

#### Orcinol reaction

This method was adapted from Kabat and Mayer [46]. Saponin extracts, a 1.6% aqueous orcinol solution and a 60% sulfuric acid solution were mixed in a 1:1:7.5 (v/v) proportion. The resulting solution was incubated in a water bath at 80°C for exactly 15 min, then cooled in tap water to stop the reaction, and its absorbance was measured at 540 nm. A 10 mg/mL solution of D-xylose (Merck, Darmstadt, Germany) in water was serially diluted to make a standard curve.

## Figures and Tables

**Table 1:** Saponins detected in the body wall (BW) and the Cuvierian tubules (CT) of five species of Holothuriidae (S: molecular structure; MW: molecular weight). Molecules indicated by crosses in bold are the major congeners observed.

Saponin name	MW	S	*Actinopyga echinites*		*Bohadschia subrubra*		*Holothuria atra*	*Holothuria leucospilota*		*Pearsonothuria graeffei*		References
				
Non-sulfated saponins			BW	CT	BW	CT	BW	BW	CT	BW	CT	
Holothurinoside C	1102	**11**								x		[19]
Desholothurin A (Nobiliside 2a)*	1118	**15**							x	x	x	[19,20]
Holothurinoside K_1_**	1134	**20**			x							
Holothurinoside E_1_	1264	**16**							x			[18]
Holothurinoside F	1410	**12**			x							[18]
Bivittoside C	1410	**22**				x						[21]
Impatienside A (Marmoratoside A)*	1424	**24**			**X**	x						[23,24]
Isomer	1424					x						
Isomer	1424					x						
Bivittoside D	1426	**23**				**X**		x	x			[21]
Isomer	1426					x						
Holothurinoside H	1440	**13**			x	x						[18]
Holothurinoside H_1_	1440	**17**			**X**	x						[18]
Isomer	1440				x							
Isomer	1440				x							
Arguside C	1442	**21**				x						[22]
Holothurinoside I	1456	**14**			x	x						[18]
Holothurinoside I_1_	1456	**18**			x	x						[18]
Isomer	1456				x							
Isomer	1456				x							
Isomer	1456				x							
Holothurinoside J_1_**	1472	**19**			x	x						

**Sulfated saponins**												
Holothurin B_3_	866	**6**	x				x	x	x			[28]
Holothurin B_1_	868	**4**	**X**				x					[26]
Holothurin B/B_4_***	882	**3**/**7**	**X**				**X**	**X**	x	x	x	[27,28]
Holothurin B_2_	884	**5**	x				x					[28]
Isomer	884		x									
Fuscocineroside B/C***	1204	**8**/**9**	x	**X**						x	x	[29]
Isomer	1204		x							x		
Isomer	1204		x									
Holothurin A_2_	1206	**2**	x							**X**	x	[26]
Isomer	1206										x	
Holothurin A	1220	**1**	x	**X**					**X**	**X**	**X**	[25]

* Different names for the same structure.

** New saponins.

*** Isomeric saponins.

**Table 2:** Saponin content in the body wall and the Cuvierian tubules of five species of Holothuriidae quantified by the measurement of the hemolytic activity and by the orcinol reaction. Values are means (±SD); numbers in brackets indicate the number of individuals tested.

Species	Body compartment	Hemolytic activity (mg eq./g*)	Orcinol reaction (mg glycoside/g)
*Actinopyga echinites*	Body wall	1.239 (1)	0.025 (1)
	Cuvierian tubules	11.359 (1)	0.278 (1)
*Bohadschia subrubra*	Body wall	1.789 (1)	0.064 (1)
	Cuvierian tubules	4.724 (1)	0.094 (1)
*Holothuria atra*	Body wall	0.973 ± 1.846 (4)	0.040 ± 0.045 (4)
*Holothuria leucospilota*	Body wall	0.324 ± 0.173 (5)	0.039 ± 0.032 (5)
	Cuvierian tubules	1.377 ± 0.864 (5)	0.040 ± 0.053 (5)
*Pearsonothuria graeffei*	Body wall	2.404 ± 0.506 (4)	0.026 ± 0.012 (4)
	Cuvierian tubules	5.361 ± 6.759 (5)	0.189 ± 0.177 (4)

* Milligram equivalents of plant saponins by gram of tissue (wet weight).
